# A Novel Statistical Method for Scene Classification Based on Multi-Object Categorization and Logistic Regression

**DOI:** 10.3390/s20143871

**Published:** 2020-07-10

**Authors:** Abrar Ahmed, Ahmad Jalal, Kibum Kim

**Affiliations:** 1 Department of Computer Science and Engineering, Air University, E-9, Islamabad 44000, Pakistan; 180159@students.au.edu.pk (A.A.); ahmadjalal@mail.au.edu.pk (A.J.); 2 Department of Human-Computer Interaction, Hanyang University, Ansan 15588, Korea

**Keywords:** adaptive weighted median filter, fuzzy c-mean segmentation, logistic regression, multiple objects categorization, multiple kernel learning, scene classification, visual sensors

## Abstract

In recent years, interest in scene classification of different indoor-outdoor scene images has increased due to major developments in visual sensor techniques. Scene classification has been demonstrated to be an efficient method for environmental observations but it is a challenging task considering the complexity of multiple objects in scenery images. These images include a combination of different properties and objects i.e., (color, text, and regions) and they are classified on the basis of optimal features. In this paper, an efficient multiclass objects categorization method is proposed for the indoor-outdoor scene classification of scenery images using benchmark datasets. We illustrate two improved methods, fuzzy c-mean and mean shift algorithms, which infer multiple object segmentation in complex images. Multiple object categorization is achieved through multiple kernel learning (MKL), which considers local descriptors and signatures of regions. The relations between multiple objects are then examined by intersection over union algorithm. Finally, scene classification is achieved by using Multi-class Logistic Regression (McLR). Experimental evaluation demonstrated that our scene classification method is superior compared to other conventional methods, especially when dealing with complex images. Our system should be applicable in various domains such as drone targeting, autonomous driving, Global positioning systems, robotics and tourist guide applications.

## 1. Introduction

Scene classification uses visual sensor technologies to explore the semantically significant information contained inside an image. Scene classification is the process of assigning categorizing labels to whole scenes based on the visual sensory data of the scene and the structure and relationships between multiple objects presented in the images. Sensors identify two broad categories (i.e., indoor and outdoor) to generally classify different scenes and these are further divided into different sub-categories based on the categories and labels pertaining to the specific multiple objects presented in the images. Visual sensors use the different properties of objects such as their local and global features to classify the whole scene. Scenery images comprise a wide variety of knowledge about the behavior of various objects which have visible features such as borders, corners, and point clouds and these enable us to learn, modify, consider alternative solutions and create new techniques to examine complex scenes. Scene interpretation [1,2] should be capable of accommodating changes in the environment being observed, identifying the vital characteristics of various objects and defining relationships among various objects in order to represent the actual scene behaviors [3,4].

Such scene information needs consistent and accurate object classification that intends to distinguish the images by evaluating semantic object properties. Object classification has become an extensively adopted field in various applications such as smart monitoring and image fetching. It also offers supplemental knowledge in the fields of activity recognition. Apart from object classification which only concentrates on limited parts of an image, scene classification is the next step that leads to scene recognition and labelling based on such limited object information [5,6]. Many scenes are comprised of complicated object relationships and, because variations among scenes can be quite subtle, accurate scene classification is a challenging task in the area of pattern matching and recognition.

The main function of scene classification is to recognize all the objects presented in the scene and to describe semantics for the accurate labeling of the whole scene. Researchers and scientists have produced a lot of work on multiple object categorization [7] for scene classification but there are still several challenges that can affect the accuracy of object categorization and recognition such as changes in illumination, the size of objects, view orientation, and occlusion between objects in complex images. Several articles [8] used a place category strategy that presents a more detailed list of the objects, summary of their spatial correlations and other static features to discriminate scenes, which affect recognition accuracy. Therefore, we propose a novel methodology, which presents the combined effects of similar region clustering, textures of objects, local/global descriptors and class distribution probability estimation. Our novel methodology produces significant performance effects compared to existing methods.

To overcome the challenges encountered in scene classification, we propose a multiple objects categorization-based method to perform scene classification of scenery images from benchmark datasets. As the first step, the proposed system preprocesses the images. We achieve efficient segmentation using two segmentation algorithms, (i) Modified Fast Super-Pixel Based Fuzzy C-Mean Segmentation (MFCS) image segmentation and (ii) Mean Shift Segmentation (MSS). In the second step the results of two algorithms are compared and analyzed. In the third step, we achieve multiple object categorization by evaluating the multiple regions detector, matching the signatures and local descriptors of the regions of images. Kernel function is used to achieve an object similarity score. Finally, the Estimated Intersection over Union (EIOU) and Multi-class Logistic Regression (McLR) are used for scene classification over challenging datasets. The main contributions of our work are as follows.

- To the best of our knowledge, this is the first time that signatures of objects, local descriptors and multiple kernel learning for objects categorization and multi-class logistic regression for scene classification have been introduced.
- Fusing of Geometric and SIFT feature descriptors for objects and scene classification.
- Accurate multiple region extraction and label indexing of complex scene datasets.
- Significant improvement in the accuracy of object and scene classification with less computational time compared to other state-of-the-art methods.

Related work is discussed in Section 2. Section 3 illustrates and details the methodology of our proposed scene classification system. Section 4 presents an analysis of our experimental results and a detailed description of the datasets. Section 5 concludes this paper.

## 2. Related Work

Exploring multiple object locations, their scale, view orientation and the impact of scenery images are challenging tasks in the visual sensors [9,10] field. We have studied the literature in several domains such as multi-object categorization, object segmentation as well as labeling and scene classification in order to establish proper parameters and metrices for our proposed method.

### 2.1. Object Segmentation

Image segmentation consists of transforming an image into a set of pixel regions represented by a mask or labels in an image. This transformation of an image into a set of pixels (a segment) allows the processing of important segments only. There are numerous techniques for the segmentation of the objects. In Sezgin et al. [11] categorized thresholding techniques into the following groups: (a) a histogram shape-based technique, (b) a clustering-based technique, (c) an entropy-based technique, (d) an object attribute-based technique, (e) a spatial method, and (f) thresholding methods. In Sujji et al. [12] discussed threshold techniques where they wanted to segment an image to detect the contours of tumors in the brain. In Bi et al. [13] proposed a segmentation method according to the fusion of motion, color and stereo cues of objects. In Yan et al. [14] proposed k-means clustering based on color image enhancement for the segmentation of cells. They computed the gray value components of R, G, and B distributions to find the mean value of these distributions. Additionally, they used YCbCr color space to represent the three clusters, achieved by dividing the improved color images. In Kamdi et al. [15] explained region growing algorithms for segmentation by comparing advantages and disadvantages. Moreover, they divided the image into regions of similar pixels by mean and by min-max techniques. In K-means clustering, the number of k segments is defined to partition the image into k groups. K groups are formed based on the similarity of color intensity or on the minimum variance from the centroid to the target pixel.

### 2.2. Single/Multiple Object Categorization

The object categorization field opens a lot of challenges for researchers in the form of finding the location of each object, identifying and describing the interactions among objects, identifying occluding objects, and delineating groups for meaningful outcomes. In Wong et al. [16] proposed an algorithm for detecting an object online and a classification of the various objects in the image. They suggested fast tracking all the objects in the scene via kernel learning instead of depending on prior knowledge of the specific object. Their implementation was performed on a Neovision2 tower benchmark dataset, which was a biologically inspired implementation that determined the shape and the movement of an object. In Sumbul et al. [17] devised the methods which included the attention of a multisource region network that calculated the pre-source feature illustration and assigned attention scores to member regions tested around the demanded object positions by utilizing their representations. They used multispectral techniques that achieved accuracies up to 64.2%. In Martin et al. [18] designed a Bayesian inference model to examine prior knowledge of each object for multiple object tracking. Then, it updated the possible mass function for closer object discrimination and applied a rate of convergence for correct classification. In Lecumberry et al. [19] computed a shape similarity measure and the steepest descent minimization method for modeling each object’s shape iteration. They used energy optimization for the automatic classification of multiple objects.

### 2.3. Scene Classification

Similarly, scene classification is a domain that provides new directions such as complicated scene contents/labels due to major ambiguities [20], similar objects properties among different scenes, and multi-instance learning in confused scenes. In Shi et al. [21] proposed a context-based saliency detection algorithm that marks saliency regions in images. They used a CNN model to construct feature points tested on five datasets, i.e., LabelMe, UIUC-Sports, Scene-15, MIT67, and SUN which produced effective results only with indoor scenes. In Zhang et al. [22] proposed the MVFL-VC method along with labeled object categorization algorithms. On the other hand, a mapping function was used to find the correlation with their labels in images. In Zhou et al. [23] proposed a simple method for indoor-outdoor scene classification, which included a bag-of-features model to construct multiple resolution images and highlighted it with dense regions. Then, partition modalities were used to produce better results for scene classification.

In Hayat et al. [24] introduced an indoor scene categorization method based on large-scale spatial layout, scale variations and rich feature descriptors for multiple distinct objects. In addition, tailored feature representations were learned by a Convolution Neural Network to effectively adopt large-scale classification. In Zou et al. [25] proposed an effective scene classification approach where fusion of local/global spatial features were adopted as collaborative representation. These features were processed by multiscale completed local binary patterns, Gabor features and SIFT patterns. Finally, they implemented Kernel collaborative classification for scene discrimination. In Ismail et al. [26] proposed a method consisting of two steps for indoor scene classification. Initially, spatial layout estimation was performed to estimate three orthogonal vanishing points and then the relationships between scene elements were represented by a layout estimation method to retrieve a high scene classification score.

## 3. Overview of Solution Framework

**Figure 1:**
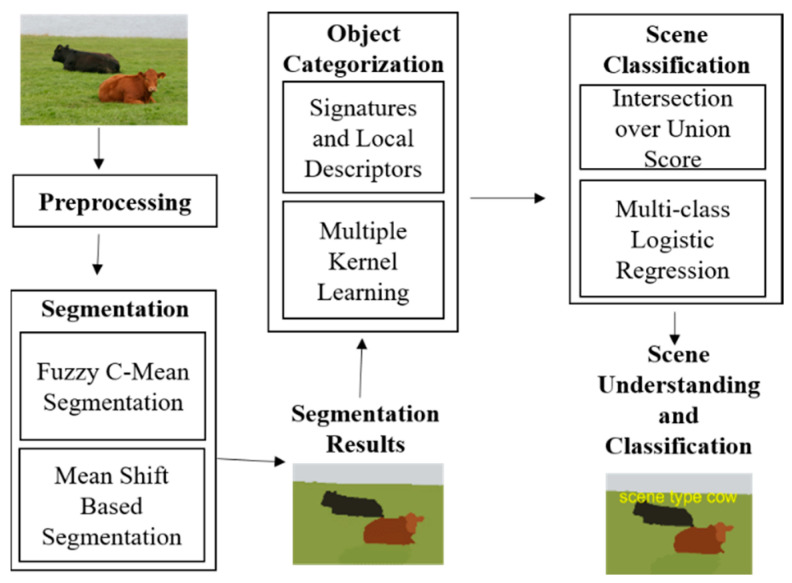
Overview of the proposed scene classification system using Multi-class Logistic Regression.

In this section, we propose a novel scene classification approach along with object categorization that accurately recognizes and labels all target objects presented in the scene. The proposed scene classification system starts with preprocessing and clearing unwanted information such as noise contents and with the normalization of object sizes for all images in the datasets. Then, the extracted data are applied to accurate object segmentation based on two distinct segmentation algorithms: modified fast super-pixel based fuzzy c-means clustering and mean shift segmentation algorithms. Multiple objects categorization is performed by considering multiple kernel learning. Finally, the proposed system achieves scene classification by using the EIOU score and McLR. Figure 1 presents an overview of the proposed scene classification system.

### 3.1. Preprocessing and Normalization

**Figure 2:**
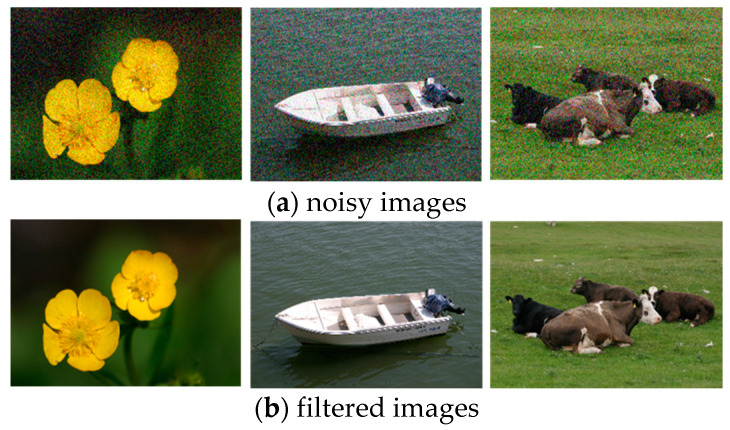
Some preprocessing steps include, (**a**) depicting noisy images and (**b**) filtering noise on images over the MSRC dataset.

During preprocessing, images are captured under different conditions such as various lights and environments which produce noise and high intensity values in the images (see Figure 2a). Therefore, to solve these issues, an Adaptive Weighted Median Filter (AWMF) [27] is applied. Such filters use an M×N sliding window which slides over all the images. It uses the local statistic weights of the image for the filtering process. The relative weights $W_{i,j}$ of the pixels (*i*, *j*) are calculated as:

(1) $$W_{i,j}=W_{0}-\frac{{aDV}_{xy}^{2}}{U_{xy}}$$

where $W_{0}$ indicates the weight of the central pixel of the frame of the filter (i.e., 3×3 or 5×5), “*a*” is the scaling factor used for the scale of frame of the filter (i.e., 3 or 5) and D is Euclidean distance between pixels.$\;U_{x,y}$ and $V_{x,y}$ are the mean and variance of the M×N sliding window respectively. $U_{x,y}$ and $V_{x,y}$ are achieved as follows:

(2) $$\;U_{x,y}=\frac{1}{MN}\overset{M-1}{\underset{i=0}{\sum }}\overset{N-1}{\underset{j=0}{\sum }}x_{i,j}$$

(3) $$V_{x,y}=\frac{1}{MN}-1\overset{M-1}{\underset{i=0}{\sum }}\overset{N-1}{\underset{j=0}{\sum }}x_{i,j}-U_{i,j}$$

Figure 2 demonstrates the preprocessing steps which include both noisy images and filtered images.

### 3.2. Single/Multiple Object Segmentation

This section provides a detailed description of single/multiple object segmentation. Object segmentation is a process in which an image is split into multiple regions. Segmentation can be achieved according to similarities in pixels or colors in a scene. As different scenes contain multiple regions, the delineation or demarcation of these regions through segmentation is a significant but challenging process in scene classification. Accuracy in segmentation greatly influences accuracy and consistency in scene classification. Images are segmented into multiple regions which are labeled with different colors. To process object segmentation, two robust segmentation methods are considered as, (i) Modified fast super-pixel based fuzzy c-means clustering image segmentation (MFCS) and (ii) mean shift segmentation (MSS).

#### 3.2.1. Modified Fast Super-pixel Based Fuzzy C-Mean Segmentation (MFCS)

Using the MFCS clustering algorithm, we achieved improved color image segmentation results compared to conventional FCM [28] methods. At the start of the process, overlapping elements are identified and pixels are taken as data points similar to the clustering approach. Then, each pixel that reveals fuzzy logic is considered to belong to more than one cluster rather than to just one defined cluster. The MFCS achieves the segmentation of the image by minimizing the objective function during iterations. In addition, these elements restrict optimal clusters of images by minimizing the weights within the clusters through a squared error objective function $J_{M}\left(U,V\right)$ which is formulated as:

(4) $$J_{M}\left(U,V\right)=\overset{c}{\underset{i=1}{\sum }}\overset{n}{\underset{j=1}{\sum }}u_{ij}^{r}|x_{j}-v_{i}{|}^{2}$$

where c represents the number of clusters, n is the data points having r any real numbers in $i^{th}$ cluster which show the fuzziness of the resulting cluster, $u_{ij}^{r}$ represents the membership of $x_{j}$ pixels of data in the $i^{th}$ cluster and $v_{i}$ which shows the cluster center:

(5) $$u_{ij}=\frac{1}{\sum_{k=1}^{c}{\left(\frac{\left|x_{j}-v_{i}\right|}{|x_{j}-v_{k}{|}^{2}}2\right)}^{\frac{1}{r-1}}}$$

(6) $$u_{ij}\in \left[0,1\right],\;\mathrm{for}\;i=\left[1,\ldots ,c\right]$$

(7) $$v_{i}=\frac{\sum_{j=1}^{n}u_{ij}^{r}x_{j}}{\sum_{j=1}^{n}u_{ij}^{r}}$$

**Figure 3:**
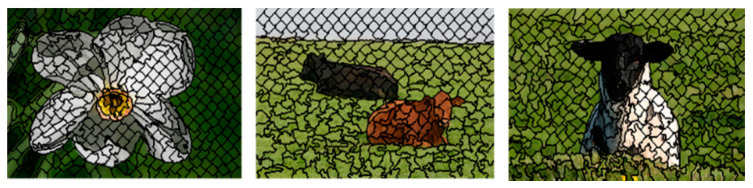
A few examples of super pixel-based pre-segmentation.

$J_{M}\left(U,V\right)$ is used to measure the distance between the corresponding pixel and the cluster center. The corresponding pixel is assigned with high value of membership when the distance between the pixel and the cluster center is minimum. The conventional FCM algorithm works on the local spatial information of pixels in images such that all neighboring regions of pixels cause high computation complexity due to analysis of spatial values at each iteration. Therefore, the proposed algorithm uses super pixel-based pre-segmentation [29] and density-based spatial clustering with noise (DBSCN) to decrease the computational complexity of Conventional FCM. Figure 3 presents the results of super pixel-based pre-segmentation. The proposed method achieved the segmentation of the color image in a few seconds on the MatLab platform running on an Intel(R) CPU 2.5 GHz core-i5 CPU 2.5 GHz and 8 GB of RAM (Intel, Santa Clara, CA, USA).

The set of data points are shown as $x_{i}=x_{1},\ldots ,x_{n}$, and $v_{i}=v_{1},\ldots ,v_{c}$ shows the set of cluster centers and *r* (any real numbers) shows the fuzziness of resulting clusters. The proposed MFCS, Algorithm 1, is carried out in steps, and the pseudo code of the MFCS algorithm is given as follows:
**Algorithm 1.** Pseudo code of the MFCM Algorithm1: Initialize the clusters c randomly 2: calculate the centers $v_{i}$ of clusters c 3: while minimum value of objective function $\;J_{M}\left(U,V\right)$ do 4:   for each data point in an image do 5:    Step 1. Measure the membership $u_{ij}$ of given data point to clusters c 6:    Step 2. Update the cluster centers $v_{i}$ 7:   end for 8: end while

**Figure 4:**
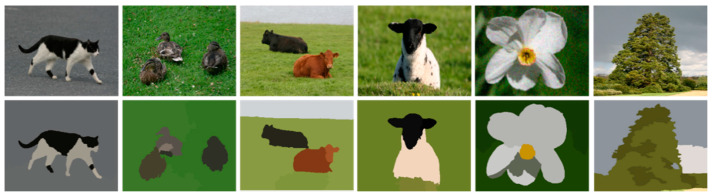
Single and multiple objects segmentation using MFCS. The 1st row shows the original while the 2nd row shows the segmentation results.

Figure 4 presents the results of the proposed MFCS algorithm over MSRC dataset.

#### 3.2.2. Mean Shift-Based Segmentation (MSS)

**Figure 5:**
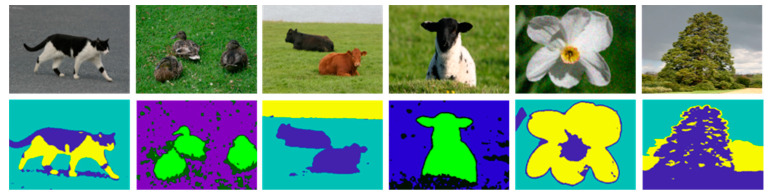
Results of Mean shift-based segmentation. The 1st row shows the original while the 2nd row shows the segmentation results.

The proposed system achieves the segmentation of an image in multiple regions using the Mean Shift Segmentation [30] algorithm. The MSS algorithm searches for the highest concentration of similar pixels space in the sample image and estimates the local density of pixels. MSS then performs density estimation iteratively and finds the minimum local value for density [31] so that all pixels having local density near to local minimum density are easily shifted to clusters of similar attributes (see Figure 5). This is a non-parametric clustering technique which does not depend on any prior knowledge of the objects or picture elements. Therefore, it can find cluster centers quickly and perform efficient object segmentation. Meanwhile, the proposed system uses kernel density estimation to find the minimum local value of density. Such kernel density $k_{E}\left(x\right)$ of window function is estimated at D dimensional space $S^{D}$ for n pixels $x_{j}$, j=1, 2, 3, … , n at a location of x can be determined as:

(8) $$k_{E}\left(x\right)=\frac{1}{n}\overset{n}{\underset{j=1}{\sum }}\frac{1}{h_{n}^{D}}k\left(\frac{x-x_{j}}{h_{x_{j}}}\right)$$

where $h_{x_{j}}$ is the width of kernel density (window function) which can be determined as:

(9) $$h\left(x_{j}\right)=h\times \left(1-d\left(x_{j}\right)\right)$$

where $d\left(x_{j}\right)$ is probability density function of given pixels space and h is a constant. Kernel density (window function) K(x) satisfies the given condition as:

(10) $$\int_{s}k\left(x\right)dx=1$$

(11) $$\int_{S^{D}}xk\left(x\right)dx=0$$

**Figure 6:**
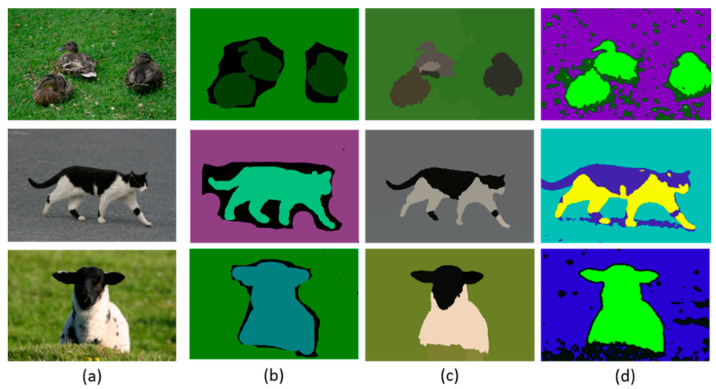
Comparison of objects segmentation examples images; (**a**) original images, (**b**) ground truth (**c**) MFCS results and (**d**) MSS results over MSRC dataset.

Thus, the proposed system analyses the results of MFCS and MSS algorithms with respect to segmentation accuracies along with ground truths and computation time efficiency. MFSC takes less computation time and produces clearer results compared to MSS. MFCS performance is more significant and better than MSS, therefore we used MFCS results for further experiments. Figure 6 indicates the comparison between the MFCS and MSS. The segmentation accuracies are evaluated by comparing the results with given ground truths of all classes from the dataset. Evaluation is carried out on the basis of pixels of segmented objects and ground truths. Table 1 indicates segmented object accuracies after comparing them with the ground truth labels.

On the other hand, Table 2 and Table 3 define the total computational time of the proposed method such as MFCS and MSS algorithms over MSRC and Corel-10k datasets, respectively.

### 3.3. Object Categorization

In this section, the proposed system used the Multiple Kernel Learning (MKL) method [32] to achieve multiple object categorization based on multiple regions and signatures of the regions in complex scenes. In object categorization, an image j (containing clusters c of multiple objects represented by different colors obtained by the segmentation process) is initially set for local descriptor $D_{j}$ (i.e., SIFT, HOG) and defines the region *R* of the image *j*. The signature $x_{j}$ is computed using a function $f_{R}$ from local descriptors $D_{j}$ as $f_{R}:D_{j}\to x_{j}$. This conversion of $f_{R}$ is mathematically derived as follows:

(12) $$Cen_{c}=\frac{1}{\left|c\right|}\sum_{j}\sum_{i}D_{icj}$$

(13) $$\mu_{c}=\frac{1}{\left|c\right|}\sum_{j}\sum_{i}\left(D_{icj}-Cen_{c}\right){\left(D_{icj}-Cen_{c}\right)}^{⊤}$$

(14) $$\mu_{j,c}=\sum_{i}\left(D_{icj}-Cen_{c}\right){\left(D_{icj}-Cen_{c}\right)}^{⊤}-\mu_{c}$$

where $Cen_{c}$ is used for the center of clusters c, |c| represents the total descriptors in the clusters c of all the images of a class, descriptors of image j that belong to cluster c are shown as $D_{icj}$ and $\mu_{c}$ represents the mean of centered descriptors that belong to clusters c. $\mu_{j,c}$ represents the computation of the signature of an image j. Then $\mu_{j,c}$ is converted into a vector $vec_{j,C}$. The signature vector $x_{j}$ of image j for all clusters c is computed by the concatenation of all $vec_{j,C}$

(15) $${vec}_{j}=\left({vec}_{j,1}\ldots {vec}_{j,C}\right)$$

**Figure 7:**
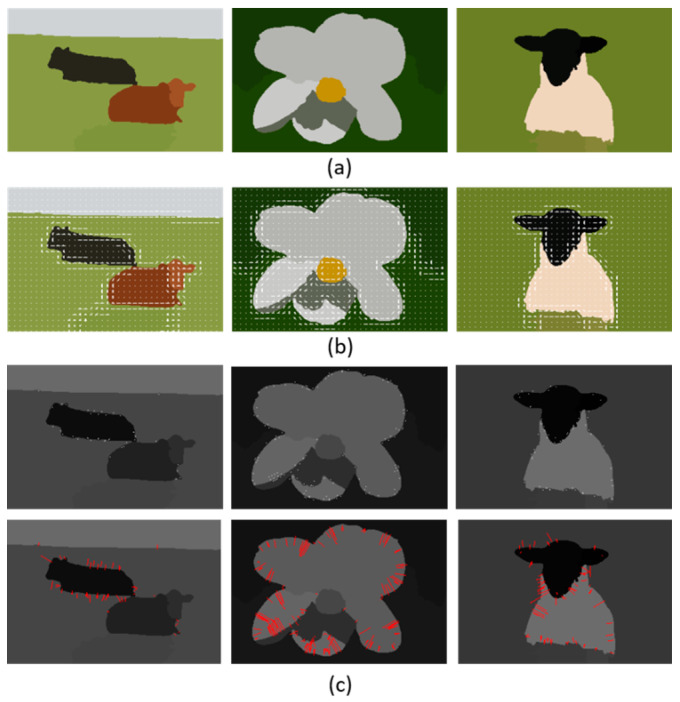
Local feature descriptor, (**a**) original images, (**b**) HOG feature extraction and (**c**) SIFT feature extraction results over MSRC dataset. The 1st row shows the locations while the 2nd row shows the scale and orientation of key points.

Figure 7 indicates the results of HOG and SIFT descriptors. These descriptors of defined region *R* are operated using a deformable parts model [33]. It produces multiple regions by drawing rectangular bounding boxes [34] over the images. The proposed system only uses bounding box regions with maximum scores given by the detector. These rectangular bounding boxes are used to indicate the regions of different foreground objects.

After defining accurate regions R of objects within the image, similarity based on the signature (extracted vectors) of this region R in *i* and *j* images is measured using kernel function $k_{R}$ as:

(16) $$k_{ℛ}\left(i,j\right)=\langle f_{R}\left(D_{Ri}\right),f_{R}\left(D_{Rj}\right)\rangle$$

**Figure 8:**
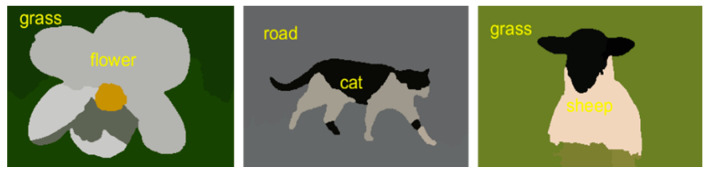
Results of object categorization using multiple kernel learning.

However, an image holds multiple regions to achieve similarity over the entire image. Therefore, the proposed system computes similarity as:

(17) $$k\left(i,j\right)=\underset{ℛ}{\sum }\omega_{ℛ}k_{ℛ}\left(i,j\right)$$

where $\omega_{ℛ}$ is associated with weights of multiple regions. Figure 8 illustrates the objects categorization method using multiple kernel learning.

### 3.4. Scene Classification

After multiple object categorization, the labeled information is further used for scene classification. This includes two significant approaches, (1) Expected Intersection over Union (EIOU) [35] score and (2) Multi-class Logistic Regression (McLR) [36]. EIOU is measured for the foreground objects and McLR is used to solve the multi-class classification problem which recognizes scenes in the images.

#### 3.4.1. Expected Intersection over Union score (EIOU)

**Figure 9:**
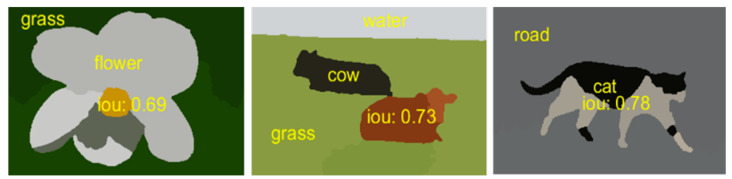
Demonstration of EIOU score over multiple objects at MSRC dataset.

The EIOU score is used to indicate how accurately we have predicted the objects and the regions of predicted objects. The EIOU score is given to all foreground objects in the images of all scenes by the proposed system and the scene is classified based on the EIOU of the foreground objects. To examine the EIOU function, we used the multiple objects $y_{j}$, their locations and the predicted objects ${\bar{y}}_{j}$. The Expected Intersection over Union $U_{EIOU}$ are achieved as follows:

(18) $$U_{EIOU}=\frac{1}{C}\overset{C}{\underset{C=1}{\sum }}U_{iou}^{\left(C\right)}\left(\bar{y},y\right)$$

where C is the number of classes and $U_{iou}^{\left(C\right)}$ is defined as:

(19) $$U_{iou}^{\left(C\right)}\;\left(\bar{y},y\right)=\frac{\sum_{j\in V}1_{\left\{{\bar{y}}_{j}-k\wedge y_{j}-C\right\}}}{\sum_{j\in V}1_{\left\{{\bar{y}}_{j}=k\vee y_{j}=C\right\}}}$$

where $y_{j}\epsilon 1,\ldots ‥,C\;\forall \;j\in V$ and V shows all pixels set in all images. $1_{\left\{{\bar{y}}_{j}-k\wedge y_{j}-C\right\}}$ represents the indicator function which gives the 1 if $\left\{{\bar{y}}_{j}-k\wedge y_{j}-C\right\}$ is true otherwise it gives 0. The ratio of the sum of pixels represents the value of $U_{iou}^{\left(C\right)}$ as the EIOU score of objects. The computed EIOU score is shown over the objects as in Figure 9.

#### 3.4.2. Multi-Class Logistic Regression (McLR)

**Figure 10:**
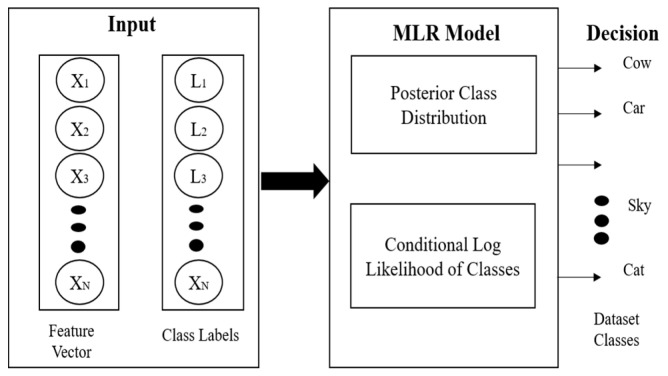
Flow architecture of multi-class logistic regression.

McLR is used for the classification of a whole scene based on multiple objects and their features. If there are multiple classes, McLR predicts the probability of given class *x* belongs to $j^{th}$(i.e., all classes of datasets). During McLR, a classifier is designed to distinguish multiple c=1,2,…K classes having *L* labeled training images using the feature vector as input. The *L* labels of all training images are $T_{L}=\left\{\left(x_{1},z_{1}\right),\ldots ,\left(x_{L},z_{L}\right)\right\}$ and the posterior class distribution (PCD) is achieved for the estimation of the $\hat{\omega }$ logistic regressor. Figure 10 shows the systematic flow of multi-class logistic regression.

The McLR is achieved as follows:

(20) $$P\left(z_{1}=c|x_{j},w\right)=\frac{\exp\left(w^{\left(c\right)}x_{j}\right)}{\sum_{c=1}^{K}\exp\left(w^{\left(c\right)}x_{j}\right)}$$

where $w^{\left(c\right)}$ is used as a logistic regressor for class c, the feature vectors are shown as $x=\left(x_{1},\ldots ,x_{j}\right)$ and set logistic regressors are shown as $w^{\left(c\right)}={\left[w_{1}^{\left(c\right)},\ldots ,\;w_{K}^{\left(c\right)}\right]}^{T}$ for class c. The posterior class probability of regressor w is achieved as follows:

(21) $$P\left(w|z_{L},x_{L}\right)\alpha p\left(z_{L}|x_{L},w\right)p\left(w|x_{L}\right)$$

**Figure 11:**
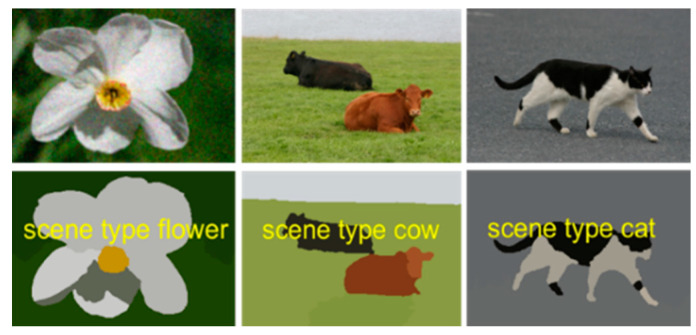
Some examples of object classification in outdoor scenes using the McLR algorithm over the MSRC object dataset.

**Figure 12:**
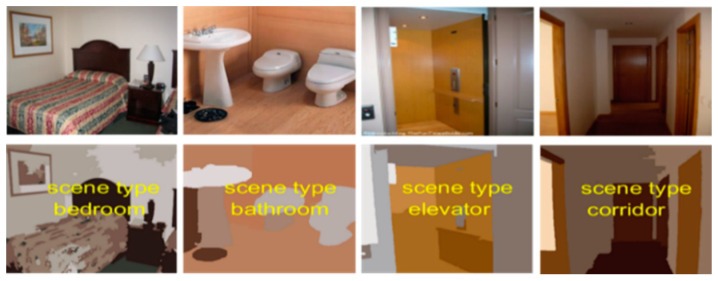
Some examples of indoor scene classification using the McLR algorithm over the CVPR 67 indoor scene dataset.

During testing, the posterior class probabilities for all feature vectors in the classes c are determined by entering the regressor into the McLR model. The class label of a feature vector is achieved by the index of the maximum posterior probability of the given test vector. The results of scene classification using McLR are shown in Figure 11 and Figure 12.

## 4. Experimental Setup and Evaluation

In this section, we present details of the experimental setup and evaluation. Object segmentation accuracy and computation time are used for performance evaluation of the proposed system for challenging indoor and outdoor datasets. We used Matlab to carry-out the experiments with a hardware system using an Intel Core i3 CPU of 2.5 GHz and 8 GB of RAM. To evaluate the performance of the proposed scene classification system, we used three different datasets: MSRC [37], Corel-10k [38] and CVPR 67 [39] datasets. For the training/testing of datasets, we used a leave-one-out-cross validation method. For the training and testing set, datasets are split into 1 and n-1 observation sets for testing and training respectively. Then, prediction weights are observed for each observation set. All the details of each dataset, their experimental results and comparisons of the proposed scene classification method with other state-of-the-art scene classification methods are given below.

### 4.1. Dataset Descriptions

#### 4.1.1. MSRC Dataset

**Figure 13:**
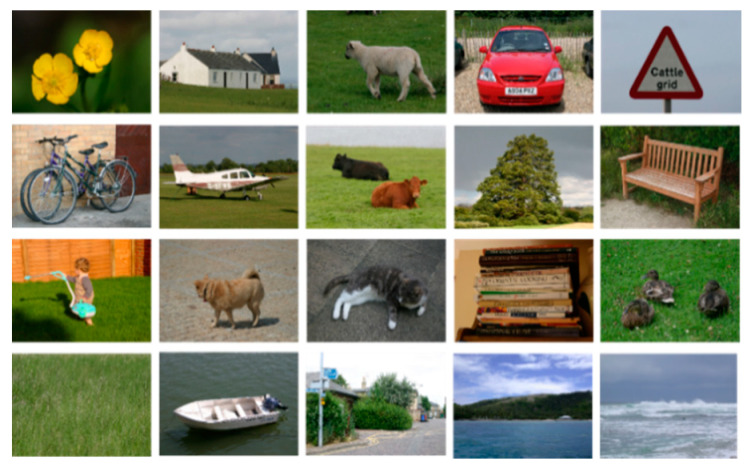
Example images from the MSRC dataset.

In the MSRC dataset, we are dealing with 591 scene images. We used twenty classes for the experimental evaluation: flower, boat, sheep, dog, car, chair, cow, bird, road, body, grass, building, sky, tree, sign, cat, water, bicycle, book and duck. Figure 13 shows example images from the MSRC dataset. Such dataset is comprised of various complicated scene images with the resolution of 213 × 320 having various objects.

#### 4.1.2. Corel-10k Dataset

**Figure 14:**
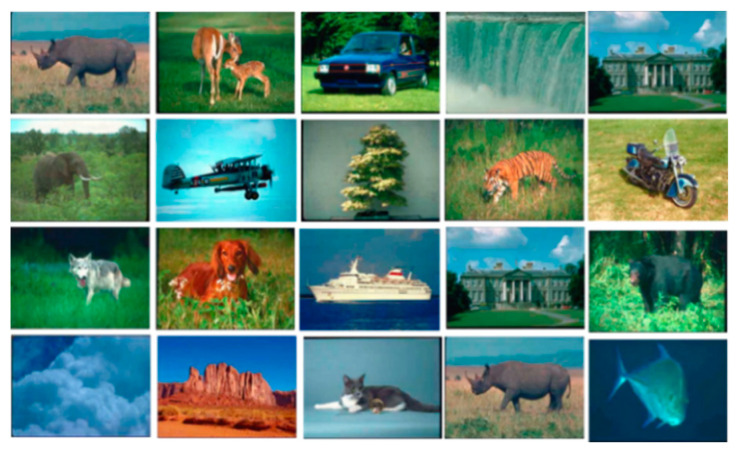
Example images from the Corel-10k dataset.

The Corel-10k dataset contains 10,000 scene images, which include multiple classes and have challenging images of different sizes and backgrounds. We performed experimental evaluations over twenty classes which included rhino, deer, car, water, building, elephant, plane, tree, tiger, bike, wolf, dog, boat, flower, bear, sky, land, cat, bird and fish. Figure 14 presents example images of the Corel-10k dataset.

#### 4.1.3. CVPR 67 indoor Scene Dataset

**Figure 15:**
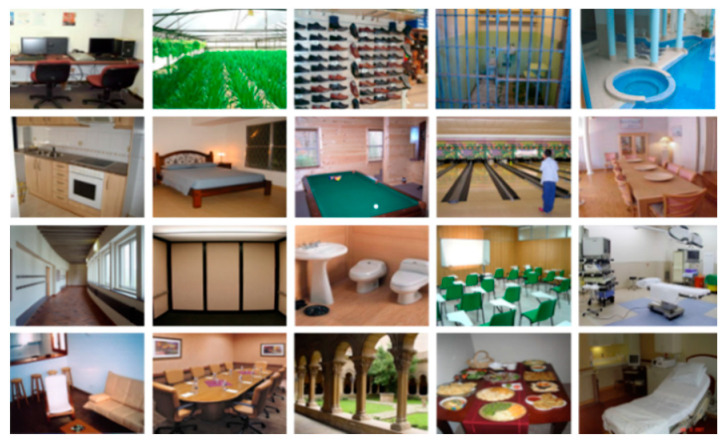
Example images from the CVPR 67 indoor scene dataset.

CVPR 67 dataset contains 67 indoor scene classes and 15,620 total images, each class consisting of 100 scene images. We performed experimental evaluation on all classes of indoor scenes (i.e., kitchen, bedroom, bathroom, corridor, elevator, locker-room, waiting-room, dining-room, game-room and garage). Figure 15 presents some example images of the CVPR 67 indoor scene dataset.

### 4.2. Experimental Results

For experiments, mean classification accuracy and comparison with existing methods were investigated by considering the indoor-outdoor scenes of all images. The proposed system achieved sufficiently informative enough results due to robust object segmentation techniques (i.e., MFCS and MSS) which reflect better performance in scene classification.

#### 4.2.1. Experiment 1: Using the MSRC Dataset

Considering the MSRC dataset, the proposed system was applied for scene classification accuracy. Table 4 shows that the major scene classes of the MSRC dataset produce remarkable performance in terms of accuracy. Table 5 summarizes the comparison of classification accuracy of the proposed method and it shows significantly better results (88.75%) than all other state-of-the-art methods. 

#### 4.2.2. Experiment 2: Using the Corel-10k Dataset

During experiments using the Corel-10k dataset, the proposed method is used with 20 different scenes and it obtained the highest classification accuracy score (85.75%) as shown in Table 6. Similarly, Table 7 shows that the proposed method has significantly higher recognition accuracy than the other state-of the-art methods such as VLAD, TNNV and LLC.

#### 4.2.3. Experiment 3: Using the CVPR 67 Indoor Scene Dataset

In the experimental evaluation using the CVPR 67 indoor scene data, the proposed method achieved scene classification accuracy of (80.02%) over 10 different classes of the CVPR 67 indoor scene dataset. The accuracy of the CVPR 67 dataset is less than the MSRC and the Corel-10k dataset caused by multiple occluded objects in different real-world scenes used in the dataset. When an object is hidden behind other objects, it is difficult to recognize it due to this occlusion effect. Table 8 shows the confusion matrix of classification using the CVPR 67 dataset.

## 5. Conclusions

In this work, we proposed a new effective scene classification system that segments single/multiple objects and classifies complex indoor-outdoor scenes. With the proposed system, object segmentation problems were explored using two robust algorithms—MFCS and MSS. In addition, object similarity was examined by multiple kernel learning. Logistic regression was used for complex scene classification. Experimental evaluations reveal that our proposed system consistently outperforms others state-of-art systems in terms of computation, segmentation and accuracy.

In future research work, we will analyze scenery images in depth to improve the accuracy of scene classification and we will work to decrease the computational complexity of scene classification. We will work in future on deep learning for indoor-outdoor scene classification to further improve classification accuracy and to expand the applicability of our work.

## Figures and Tables

**Table 1:** Objects Segmentation Accuracy of MFCS Algorithm over MSRC Dataset.

**Classes**	**fl**	**bo**	**sh**	**do**	**ca**	**co**	**Bi**
Accuracy (%)	92.3	88.6	96.4	94.6	82.7	94	87
**Classes**	**ro**	**bd**	**gr**	**ch**	**du**	**bu**	**Sk**
Accuracy (%)	83.3	86.8	89.3	79.9	88.4	84.8	87
**Classes**	**tr**	**si**	**ct**	**wt**	**bc**	**bk**	
Accuracy (%)	84.4	78.2	87.9	92	79.8	78	
Mean Segmentation Accuracy = 86.77 %							

fl = flower; bo = boat; sh = sheep; do = dog; ca = car; co = cow; bi = bird; ro = road; bd = body; gr = grass; ch = chair; du = duck; bu = building; sk = sky; tr = tree; si = sign; ct = cat; wt = water; bc = bicycle; bk = book.

**Table 2:** Comparison of Computation Time of Objects Segmentation Algorithms over MSRC Dataset.

Class	MFCS	MSS	Class	MFCS	MSS
**fl**	76.5	78.2	**ch**	84.6	98.7
**bo**	47.4	47.8	**bu**	92.3	93.4
**sh**	65.9	71.2	**sk**	32.7	35.5
**do**	35.2	43.5	**tr**	54.5	61.8
**ca**	45.8	46.1	**si**	46.7	47.0
**co**	97.5	101.5	**ct**	63.1	65.2
**bi**	41.1	43.7	**wt**	29.8	33.5
**ro**	52.6	53.1	**bc**	36.2	41.8
**bd**	39.2	42.9	**bk**	54.7	52.1
**gr**	51.4	52.2	**du**	172.9	201.5
Mean computational time of the MFCS algorithm = 61.00 s					
Mean computational time of the MSS algorithm = 65.53 s					

**Table 3:** Comparison of Computation Time for Object Segmentation Algorithms over Corel-10k Dataset.

Class	MFCS	MSS	Class	MFCS	MSS
**rh**	112.0	131.2	**wo**	130.6	149.5
**dr**	130.1	143.5	**do**	129.1	148.2
**ca**	91.7	105.0	**bo**	150	168.9
**wa**	87.4	99.3	**fl**	114.5	126.1
**bu**	171.0	188.9	**be**	145.8	166.0
**el**	96.5	114.2	**sk**	89.0	104.5
**ai**	150.2	170.3	**la**	97.5	113.2
**tr**	94.1	105.9	**ct**	122.9	143.9
**ti**	133.2	156.3	**bd**	131.2	157.0
**bi**	170.9	199.2	**fi**	135.0	162.7
Mean computational time of the MFCS algorithm = 124.13 s					
Mean computational time of the MSS algorithm = 142.69 s					

rh = rhino; dr = deer; ca = car; wa = water; bu = building; el = elephant; ai = airplane; tr = tree; ti = tiger; bi = bike; wo = wolf; do = dog; bo = boat; fl = flower; be = beer; sk = sky; la = land; ct = cat; bd = bird; fi = fish.

**Table 4:** Confusion matrix of accuracy scores for object classification in outdoor scenes for the proposed approach and the MSRC dataset.

	fl	bo	sh	do	ca	co	bi	ro	bd	gr	ch	du	bu	sk	tr	si	ct	wt	bc	Bk
**fl**	**0.95**	0	0	0	0	0	0.1	0	0	0.4	0	0	0	0	0.1	0	0	0	0	0
**bo**	0	**0.89**	0	0	0	0	0.1	0	0	0	0	0.5	0	0	0	0.1	0	0.4	0	0
**sh**	0	0	**0.92**	0.2	0	0.5	0	0	0	0	0	0	0	0	0	0	0.1	0	0	0
**do**	0	0	0.2	**0.89**	0	0	0	0	0	0	0	0	0	0	0	0	0.7	0	0	0
**ca**	0	0.2	0	0	**0.84**	0	0.7	0.5	0	0	0	0	0.3	0	0	0	0	0	0	0
**co**	0	0	0.7	0	0	**0.93**	0	0	0	0	0	0	0	0	0	0	0	0	0	0
**bi**	0	0	0	0	0	0	**0.90**	0	0	0	0	0	0	0.9	0	0	0	0.1	0	0
**ro**	0	0	0	0	0	0	0	**0.87**	0	0	0	0	0	0	0	0	0	0	0	0
**bd**	0	0	0	0	0.2	0	0	0	**0.89**	0.1	0	0	0.3	0	0	0.2	0	0	0.1	0.2
**gr**	0	0	0	0	0	0	0	0	0	**0.91**	0	0	0	0.2	0.9	0	0	0	0	0
**ch**	0	0	0	0	0.1	0	0	0.3	0	0	**0.88**	0	0.4	0	0	0.2	0	0	0	0.2
**du**	0	0	0	0	0	0	0	0	0	0	0	**0.85**	0	0	0	0	0	0	0	0
**bu**	0	0	0	0	0	0	0	0	0	0.2	0	0	**0.88**	0	0	0	0	0.9	0	0.1
**sk**	0	0	0	0	0	0	0	0	0	0	0	0	0.3	**0.87**	0	0	0	0.9	0	0.1
**tr**	0	0	0	0	0	0	0	0	0	0.9	0	0	0	0	**0.88**	0	0	0	0	0
**si**	0	0	0	0	0	0	0	0.3	0	0	0	0	0.4	0	0	**0.89**	0	0	0	0.4
**ct**	0	0	0	0	0.9	0	0	0	0	0	0	0	0	0	0	0.2	**0.88**	0	0	0.1
**wt**	0	0.2	0	0	0	0	0	0	0	0	0	0	0	0.8	0	0	0	**0.90**	0	0
**bc**	0	0	0	0	0.6	0	0	0.1	0	0	0	0	0.2	0	0	0.2	0	0	**0.89**	0
**bk**	0.1	0	0	0	0	0	0	0	0	0	0.5	0	0.2	0	0	0.8	0	0	0	**0.84**

fl = flower; bo = boat; sh = sheep; do = dog; ca = car; co = cow; bi = bird; ro = road; bd = body; gr = grass; ch = chair; du = duck; bu = building; sk = sky; tr = tree; si = sign; ct = cat; wt = water; bc = bicycle; bk = book.

**Table 5:** Comparison of the proposed method with other state-of-the art methods using the MSRC dataset.

Methods	Classification Accuracy (%)
Bayesian model [40]	82.9
Scene classification using machine performance [41]	81.0
Scene classification with weighted method [42]	84.7
Proposed Method	88.75

**Table 6:** Confusion matrix of accuracy for object classification of outdoor scenes for the proposed approach using the Corel-10k dataset.

	rh	de	ca	wt	bu	el	ai	tr	ti	bi	wl	do	bo	fl	be	sk	la	ct	bd	fi
**rh**	**0.87**	0	0	0	0	0.9	0	0	0.2	0	0.1	0.1	0	0	0	0	0	0	0	0
**de**	0.3	**0.76**	0	0	0	0.5	0	0	0.8	0	0.4	0.2	0	0	0	0	0	0.2	0	0
**ca**	0	0	**0.83**	0	0.7	0	0.6	0	0	0	0	0	0.4	0	0	0	0	0	0	0
**wt**	0	0	0	**0.91**	0.1	0	0	0	0	0	0	0	0.1	0	0	0.7	0	0	0	0
**bu**	0	0	0	0.3	**0.84**	0	0.7	0	0	0.3	0	0	0.4	0	0	0	0	0	0	0
**el**	0.5	0	0	0	0	**0.93**	0	0	0.1	0	0.1	0	0	0	0	0	0	0	0	0
**ai**	0	0	0.3	0	0.5	0	**0.90**	0	0	0	0	0	0.2	0	0	0	0	0	0	0
**tr**	0	0	0	0.2	0	0	0	**0.91**	0	0	0	0	0	0.6	0	0.1	0	0	0	0
**ti**	0.1	0	0.2	0	0	0.3	0	0	**0.89**	0	0.4	0	0	0	0	0	0	0.1	0	0
**bi**	0	0	0.9	0	0.2	0	0.4	0	0	**0.79**	0	0	0.6	0	0	0	0	0	0	0
**wl**	0	0.1	0	0	0	0.1	0	0	0.6	0	**0.88**	0.4	0	0	0	0	0	0	0	0
**do**	0	0.2	0	0	0	0	0	0	0.6	0	0.2	**0.87**	0	0	0	0	0	0.3	0	0
**bo**	0	0	0	0.4	0.5	0	0.3	0	0	0.3	0	0	**0.83**	0	0	0	0	0	0	0.2
**fl**	0	0	0	0	0	0	0	0.9	0	0.2	0	0	0	**0.84**	0	0.2	0.3	0	0	0
**be**	0.2	0	0	0	0	0.3	0	0	0.3	0	0	0.2	0	0	**0.90**	0	0	0	0	0
**si**	0	0	0	0	0	0	0	0	0	0	0	0	0	0.8	0	**0.89**	0.1	0	0	02
**sk**	0	0	0	0.9	0	0	0	0	0	0	0	0	0.1	0	0	0	**0.88**	0	0	0.2
**la**	0	0	0	0.6	0	0	0	0.4	0	0	0	0	0	0.3	0	0	0.4	**0.83**	0	0
**bd**	0	0	0	0	0	0	0.5	0	0	0	0	0	0	0.5	0	0.4	0	0	**0.83**	0.3
**fi**	0	0	0	0.9	0	0	0	0	0	0.2	0	0	0.2	0.4	0	0	0.5	0.1	0	**0.77**

rh = rhino; de = deer; ca = car; wt = water; bu = building; el = elephant; ai = airplane; tr = tree; ti = tiger; bi = bike; wl = wolf; do = dog; bo = boat; fl = flower; be = beer; sk = sky; la = land; ct = cat; bd = bird; fi = fish.

**Table 7:** Comparison of the proposed method with other state-of-the art methods using the Corel-10k dataset.

Methods	Classification Accuracy (%)
VLAD [43]	80.0
TNNVLAD [44]	81.0
VLAD + LLC [45]	83.7
Proposed Method	85.75

**Table 8:** Confusion Matrix of scene classification accuracy for the proposed approach using the CVPR 67 indoor scene dataset.

Class	Accuracy %	Class	Accuracy %	Class	Accuracy %
kitchen	0.89	grocery store	0.79	nursery	0.83
bedroom	0.85	florist	0.82	train station	0.82
bathroom	0.87	church inside	0.83	laundromat	0.79
corridor	0.76	auditorium	0.82	stairs case	0.81
elevator	0.80	buffet	0.77	gym	0.78
locker room	0.78	class room	0.81	tv studio	0.76
waiting room	0.81	green house	0.75	pantry	0.80
dining room	0.83	bowling	0.79	pool inside	0. 77
game room	0.79	cloister	0.83	inside subway	0.79
garage	0.82	concert hall	0.81	wine cellar	0.77
lobby	0.77	computer room	0.80	fast food restaurant	0.76
office	0.79	dental office	0.84	bar	0.82
mall	0.81	library	0.79	clothing store	0.81
Laboratory wet	0.77	inside bus	0.77	casino	0.83
jewelry shop	0.79	closet	0.81	deli	0.79
museum	0.82	studio music	0.79	book store	0.80
living room	0.77	lobby	0.80	children room	0.82
movie theater	0.83	prison cell	0.84	hospital room	0.79
toy store	0.80	hair saloon	0.80	kinder garden	0.77
operating room	0.82	subway	0.81	shoe shop	0.76
airport inside	0.79	warehouse	0.77	restaurant kitchen	0.78
art studio	0.80	meeting room	0.82	bakery	0.79
video store	0.76				
Mean Scene Classification Accuracy = 80.02 %					

## References

[1] Liu Y., Gu Y., Yan F., Zhuang Y. (2019). Outdoor Scene Understanding Based on Multi-Scale PBA Image Features and Point Cloud Features. Sensors.

[2] Chen C., Li S., Fu X., Ren Y., Chen Y., Kuo C.C.J. Exploring confusing scene classes for the places dataset: Insights and solutions. Proceedings of the Asia-Pacific Signal and Information Processing Association Annual Summit and Conference.

[3] Chen L., Cui X., Li Z., Yuan Z., Xing J., Xing X., Jia Z. (2019). A New Deep Learning Algorithm for SAR Scene Classification Based on Spatial Statistical Modeling and Features Re-Calibration. Sensors.

[4] Chen C., Ren Y., Kuo C.C.J. (2016). Outdoor scene classification using labeled segments. In Big Visual Data Analysis.

[5] Susan S., Agrawal P., Mittal M., Bansal S. (2019). New shape descriptor in the context of edge continuity. CAAI Trans. Intell. Technol..

[6] Chen C., Ren Y., Kuo C.C.J. Large-scale indoor/outdoor image classification via expert decision fusion (edf). Proceedings of the Asian Conference on Computer Vision.

[7] Zhang C., Cheng J., Li L., Li C., Tian Q. (2017). Object categorization using class-specific representations. IEEE Trans. Neu. Net. Learn. Sys..

[8] Rafique A.A., Jalal A., Ahmed A. Scene Understanding and Recognition: Statistical Segmented Model using Geometrical Features and Gaussian Naïve Bayes. Proceedings of the IEEE conference on International Conference on Applied and Engineering Mathematics.

[9] Jalal A., Kamal S., Kim D. (2014). A depth video sensor-based life-logging human activity recognition system for elderly care in smart indoor environments. Sensors.

[10] Shokri M., Tavakoli K. (2019). A review on the artificial neural network approach to analysis and prediction of seismic damage in infrastructure. Int. J. Hydromechatron..

[11] Sezgin M., Sankur B. (2004). Survey over image thresholding techniques and quantitative performance evaluation. J. Elect. Imaging.

[12] Sujji G.E., Lakshmi Y.V.S., Jiji G.W. (2013). MRI brain image segmentation based on thresholding. Int. J. Adv. Comput. Res..

[13] Bi S., Liang D. (2006). Human segmentation in a complex situation based on properties of the human visual system. Intell. Control Autom..

[14] Yan M., Cai J., Gao J., Luo L. K-means cluster algorithm based on color image enhancement for cell segmentation. Proceedings of the 5th International Conference on BioMedical Engineering and Informatics.

[15] Kamdi S., Krishna R.K. (2012). Image segmentation and region growing algorithm. Int. J. Comput. Tecnol. Elect. Eng..

[16] Wong S.C., Stamatescu V., Gatt A., Kearney D., Lee I., McDonnell M.D. (2017). Track everything: Limiting prior knowledge in online multi-object recognition. IEEE Trans. Image Proc..

[17] Sumbul G., Cinbis R.G., Aksoy S. (2019). Multisource Region Attention Network for Fine-Grained Object Recognition in Remote Sensing Imagery. IEEE Trans. Geosci. Remote Sens..

[18] Martin S. Sequential bayesian inference models for multiple object classification. Proceedings of the 14th International Conference on Information Fusion.

[19] Lecumberry F., Pardo A., Sapiro G. Multiple shape models for simultaneous object classification and segmentation. Proceedings of the 16th IEEE International Conference on Image Processing.

[20] Jalal A., Kim Y.H., Kim Y.J., Kamal S., Kim D. (2017). Robust human activity recognition from depth video using spatiotemporal multi-fused features. Pattern Recognit..

[21] Shi J., Zhu H., Yu S., Wu W., Shi H. (2019). Scene Categorization Model Using Deep Visually Sensitive Features. IEEE Access.

[22] Zhang C., Cheng J., Tian Q. (2019). Multiview, Few-Labeled Object Categorization by Predicting Labels with View Consistency. IEEE Trans..

[23] Zhou L., Zhou Z., Hu D. (2013). Scene classification using a multi-resolution bag-of-features model. Pattern Recognit..

[24] Hayat M., Khan S.H., Bennamoun M., An S. (2016). A Spatial Layout and Scale Invariant Feature Representation for Indoor Scene Classification. IEEE Trans. Image Proc..

[25] Zou J., Li W., Chen C., Du Q. (2016). Scene classification using local and global features with collaborative representation fusion. Inf. Sci..

[26] Ismail A.S., Seifelnasr M.M., Guo H. Understanding Indoor Scene: Spatial Layout Estimation, Scene Classification, and Object Detection. Proceedings of the 3rd International Conference on Multimedia Systems and Signal Processing.

[27] Tingting Y., Junqian W., Lintai W., Yong X. (2019). Three-stage network for age estimation. CAAI Trans. Intell. Technol..

[28] Mahajan S.M., Dubey Y.K. Color image segmentation using kernalized fuzzy c-means clustering. Proceedings of the 2015 Fifth International Conference on Communication Systems and Network Technologies.

[29] Zhu. C., Miao D. (2019). Influence of kernel clustering on an RBFN. CAAI Trans. Intell. Technol..

[30] Gandhi N.J., Shah V.J., Kshirsagar R. Mean shift technique for image segmentation and Modified Canny Edge Detection Algorithm for circle detection. Proceedings of the 2014 International Conference on Communication and Signal Processing.

[31] Wiens T. (2019). Engine speed reduction for hydraulic machinery using predictive algorithms. Int. J. Hydromechatron..

[32] Durand T., Picard D., Thome N., Cord M. Semantic pooling for image categorization using multiple kernel learning. Proceedings of the 2014 IEEE International Conference on Image Processing.

[33] Felzenszwalb P.F., Girshick R.B., McAllester D., Ramanan D. (2009). Object detection with discriminatively trained part-based models. IEEE Trans. Pattern Anal. Mach. Intell..

[34] Osterland. S., Weber J. (2019). Analytical analysis of single-stage pressure relief valves. Int. J. Hydromechatron..

[35] Nowozin S. Optimal decisions from probabilistic models: The intersection-over-union case. Proceedings of the IEEE Conference on Computer Vision and Pattern Recognition.

[36] Behadada O., Trovati M., Chikh M.A., Bessis N., Korkontzelos Y. Logistic regression multinomial for arrhythmia detection. Proceedings of the 2016 IEEE 1st International Workshops on Foundations and Applications of Self* Systems (FAS*W).

[37] Shotton J., Winn J., Rother C., Criminisi A. Textonboost: Joint appearance, shape and context modeling for multi-class object recognition and segmentation. Proceedings of the European Conference on Computer Vision.

[38] Liu G.H., Yang J.Y., Lo Z.Y. (2015). Content-based image retrieval using computational visual attention model, Pattern Recognition. Pattern Rec..

[39] Quattoni A., Torralba A. Recognizing indoor scenes. Proceedings of the 2009 IEEE Conference on Computer Vision and Pattern Recognition.

[40] Irie G., Liu D., Li Z., Chang S.F. A bayesian approach to multimodal visual dictionary learning. Proceedings of the 2013 IEEE Conference on Computer Vision and Pattern Recognition.

[41] Mottaghi R., Fidler S., Yuille A., Urtasun R., Parikh D. (2015). Human-machine CRFs for identifying bottlenecks in scene understanding. IEEE Trans. Pattern Anal. Mach. Intell..

[42] Liu X., Yang W., Lin L., Wang Q., Cai Z., Lai J. (2015). Data-driven scene understanding with adaptively retrieved exemplars. IEEE Multidiscip..

[43] Jegou H., Perronnin F., Douze M., Sánchez J., Perez P., Schmid C. (2011). Aggregating local image descriptors into compact codes. IEEE Trans. Pattern Anal. Mach. Intell..

[44] Long X., Lu H., Peng Y., Wang X., Feng S. (2016). Image classification based on improved VLAD. Multimed. Tools Appl..

[45] Cheng C., Long X., Li Y. VLAD Encoding Based on LLC for Image Classification. Proceedings of the 2019 11th International Conference on Machine Learning and Computing.

